# The value of mortality data to advance learning and decision-making dynamics in Mozambique’s health system

**DOI:** 10.1186/s13690-026-02044-5

**Published:** 2026-08-14

**Authors:** Amilcar Magaço, Fabrizio Tediosi, Inácio Mandomando, Mirko S. Winkler, Khátia Munguambe, Daniel Cobos Muñoz

**Affiliations:** 1 Swiss Tropical and Public Health Institute, Basel, Switzerland; 2 University of Basel, Basel, Switzerland; 3 Centro de Investigação em Saúde de Manhiça, Maputo, Moçambique; 4 University of Milan, Milan, Italy; 5 Global Health and Tropical Medicine, GHMT, LA-REAL, Instituto de Higiene e Medicina Tropical, IHMT, Universidade NOVA de Lisboa, Lisboa, Portugal; 6 ISGlobal, Barcelona, Spain; 7 Instituto Nacional de Saúde, Marracuene, Moçambique; 8 Faculty of Medicine, University Eduardo Mondlane, Maputo, Mozambique

**Keywords:** Mortality data, Learning, Decision-making, Health system, Mozambique

## Abstract

**Introduction:**

There is a lack of evidence on whether and how health systems stakeholders in Mozambique learn from routine health data. Despite the recurring claim by the stakeholders themselves that data in general should be the centre of informed public health policy, it remains unclear how decision-makers and managers access, interpret, and use mortality data for learning and decision-making. Using the Learning Health System (LHS) lens, we examined how mortality data are accessed, interpreted, and used across system levels in Mozambique.

**Methods:**

We conducted a qualitative case study from September to November 2024, involving 20 key informants from the Ministry of Health at both national and subnational levels, non-governmental organisations, data producers, and users. Data were collected in the districts of Manhiça and Quelimane in Mozambique, through semi-structured interviews, transcribed, coded, and thematically analysed using NVivo12, guided by the LHS dimensions: learning levels, learning loops, and means of learning.

**Results:**

Participants recognised the value of mortality data for learning and decision-making, but routine use remained uneven. At the central Ministry of Health level, mortality data informed strategic adjustment and emerging double-loop learning, particularly through reprioritisation, planning, and resource reallocation discussions. At the subnational level, mortality data mainly supported single-loop learning, where routine reports and outbreak signals triggered immediate adjustments in clinical practice and public health response. Delays in data access, low data literacy among decision-makers, fragmented information systems, and centralised decision-making appear to be the greatest challenges to the effective use of mortality data. Budget authority also remains centralised, and allocation decisions are largely locked into the wage bill rather than the system’s needs in terms of functionality. Heavy dependence on central directives limits local autonomy and its ability to act, preventing new ways of learning and working.

**Conclusion:**

Mortality data already support operational and strategic learning in Mozambique, particularly through single- and double-loop processes. Systemic transformation in how the system learns remains limited and is exacerbated by structural barriers within the health system. Priorities include improving timely access to mortality information across levels, strengthening analytical and interpretive capacity, integrating fragmented systems, and widening delegated decision space, which will ultimately enhance evidence-based planning, local action, and health system learning.

**Supplementary Information:**

The online version contains supplementary material available at https://doi.org/10.1186/s13690-026-02044-5.

**Table:** 

Text box 1. Contributions to the literature
This study contributes to the literature in four key ways:
• Mortality data are a key measure of population health. This study shows how such data are often produced without being used to guide routine decisions.
• This study adds evidence from Mozambique on how mortality data move, or fail to move, from reporting to discussion and action across health system levels.
• It applies a Learning Health System lens to show where the data-to-decision pathway breaks (timeliness, interpretation skills, fragmented sources, and limited local decision space).
• It highlights practical system steps to strengthen population health policy use of mortality data, beyond investing only in data collection.

## Introduction

The need for evidence-based decision-making in the health sector has been widely recognised for several decades across the global health community [1]. When used properly, enables health systems to prioritise challenges, allocate resources efficiently, design targeted interventions, and monitor progress to improve health outcomes [2, 3]. However, translating data into actionable policy and practice remains a major challenge in many Low- and Middle-Income Countries (LMICs), is made harder by recent contractions in external health financing (e.g., a 21% global Development Assistance for Health (DAH), decline between 2024 and 2025, largely from reduced U.S. funding) and replenishment shortfalls in major global health mechanisms [4].

The process by which health systems learn has been a subject of study for decades. The Learning Health System (LHS) approach frames a health system as one that can routinely learn from the evidence it generates, moving through cycles of information, deliberation, and action to improve outcomes, embedding learning as a core function rather than a one-off activity [5]. Global health actors have increasingly promoted this approach to strengthen feedback loops between data, dialogue, and decisions. This is especially relevant for LMICs, where persistent gaps in performance, accountability, and equity demand systemic approaches such as strengthening data governance, developing analytical and interpretive capabilities, and establishing formal forums and decision-making processes that use evidence to align incentives and decision-making processes. Yet, as recent literature notes, these efforts yield benefits only when learning is embedded across the system and health information systems (HIS) are coupled to mechanisms for reflection and strategic change [6, 7]. In this context, mortality data should function not only as outcome indicators but also as catalysts for learning and decision-making; however, this potential is not yet fully realised in many LMICs [5, 8, 9], including Mozambique.

Nonetheless, in many LMICs, incomplete civil registration and vital statistics, and fragmented routine HIS mean large shares of deaths are not being recorded or lack an assigned cause [10]. As a result, decisions are often based on inaccurate information, not only due to deficiencies in routine HIS and fragmented data systems [11]. In practice, when “data use” occurs, it tends to rely on readily available service provision and access indicators rather than mortality information, leaving the potential of mortality data to improve health outcomes largely untapped [7, 12].

In Mozambique, investments in the Health Information System for Monitoring and Evaluation (SIS-MA, developed based on DHIS2) and mortality surveillance have aimed to improve data availability and quality and to promote use for planning and resource allocation [6]. Despite these advances, routine use of mortality information remains uneven and often centralised, with few institutional mechanisms that incorporate mortality data into routine planning, resource allocation, and monitoring [13].

This qualitative case study examines how mortality data are accessed, interpreted, and used for learning and informing decision-making in Mozambique’s health system. We aim to map current practices across national and subnational levels, identify where data-to-action breaks down, and distil practical opportunities to strengthen routine use of mortality data to support more responsive, equitable, and effective decision-making to ultimately improve health outcomes.

## Methods

### Study design

We conducted a qualitative case study [14] to analyse decision-makers’ and managers’ perspectives on using mortality data for health system learning and decision-making in Mozambique. The case studied in this paper is the Mozambican health system’s use of mortality data through the lens of the LHS. The embedded units of analysis were the Ministry of Health (MoH), selected provincial/district health directorates, health facilities, and partner non-governmental organisations (NGOs) operating within the selected districts.

### Study setting

**Fig. 1:**
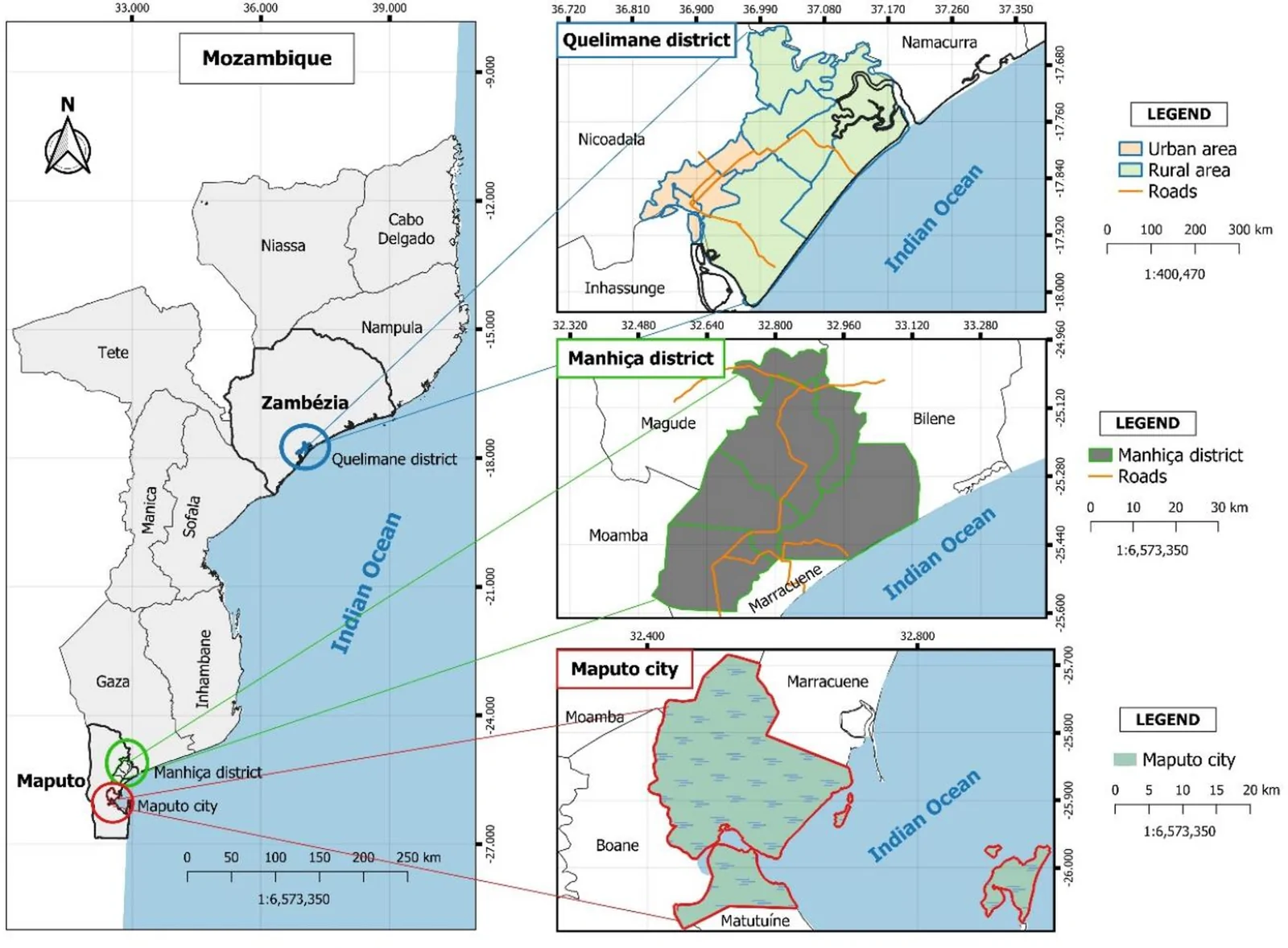
Study sites for interviews on mortality data use in Mozambique’s health system, September–November 2024

Mozambique is a southern African country with over 34 million inhabitants that is classified as a low-income country. The health system in Mozambique is organised in tiers (central, provincial, district, health facility), with the MoH headquartered in Maputo, the capital City. This study was conducted in three locations: (i) Maputo City, where central-level MoH policy and decision-makers were interviewed on the use of mortality data for learning and decision-making; (ii) Manhiça district, a predominantly rural setting in Maputo province in the south of the country; and (iii) Quelimane city, the capital of the central province of Zambézia, which includes both urban and rural areas (Fig. 1). Manhiça and Quelimane were purposively selected to gather subnational views on the use of mortality data from MoH local managers, NGOs, and healthcare providers, and because mortality surveillance systems (MSS) have operated in these two districts since 2016, capturing deaths in both health facilities and communities and providing sustained, comparable data for this study [15].

### Sampling and recruitment of study participants

To recruit participants with experience in producing or using mortality data in the health system, we used a purposive sampling strategy, complemented by snowball sampling, to identify additional participants [16]. We applied stratified purposive sampling across system levels and roles. First, a list was compiled based on the MoH departments, where 15 individuals were identified who met the following inclusion criteria: currently employed at national or subnational level in one of the three study sites; whose role involves mortality data production, management, interpretation, or use for planning/decision-making; and who have been in the position for at least 12 months. From this group, 8 individuals were recruited and interviewed, selected on eligibility and availability within the fieldwork window. After each interview, informants were asked to suggest additional information-rich participants to fill gaps in strata and perspectives. Potential participants were then contacted by phone or in person at their workplaces and invited to take part in the study. Recruitment proceeded in parallel across strata until thematic saturation was reached, meaning that no further interviews were needed. This was defined as the absence of new ideas or significantly innovative themes in the final interviews. In total, 20 interviews were conducted across all strata.

### Data collection

Data collection took place between September and November 2024, with a two-week interruption due to the presidential elections held on October 9th. The semi-structured interviews (SSI) were conducted in Portuguese by the principal investigator (AM), who is an experienced qualitative researcher. The SSI was designed inductively based on prior data-use literature in similar settings [17–19]. They consisted mainly of open-ended questions about access to and use of mortality data, decision-making windows, alignment with local partners, and the feedback loop at different levels of the health system (see Supplementary Material File S1: Annex I SSI Guides). These topics and general questions were used to understand how health systems learn from the country’s available mortality data. The SSI was conducted face-to-face in the participants’ workplace. Due to the participants’ unavailability for a face-to-face meeting, two of the interviews were conducted via Zoom. Each interview lasted approximately 30 to 45 min. All SSI were digitally recorded.

During the interviews, we recognised potential power dynamics when interviewing MoH staff and stakeholders from NGOs, as well as the risk of social desirability bias. To mitigate these risks, AM emphasised confidentiality and the independence of the study at the beginning of each interview, clarified that there were no right or wrong answers, and conducted interviews in private settings.

### Reflexivity

During data collection in the field, the researcher (AM) kept reflective notes after each interview and held regular debriefings with the co-authors, enabling questions to be refined, counter-examples to be sought, and emerging themes to be explored in greater depth. We recognised that our proximity to health information systems as researchers could lead to bias, so we used open-ended questions and neutral probing during the interviews to mitigate this. Reflexivity also guided theoretical sampling, including the addition of new profiles when gaps emerged, and code revision as new interpretations emerged. These strategies reinforced credibility and confirmability, making our LHS-guided lenses and analysis explicit.

### Data management and analysis

**Fig. 2:**
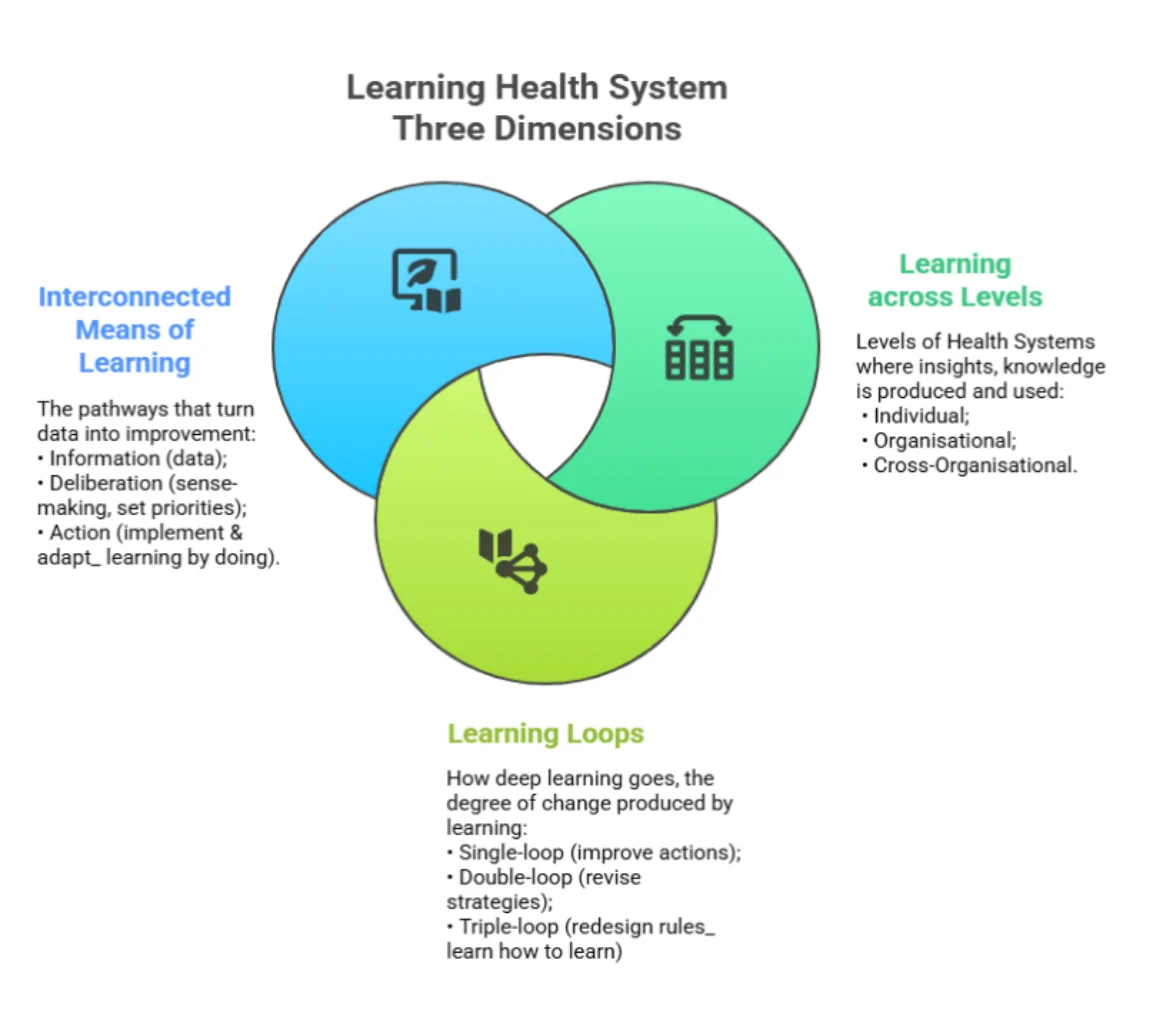
Learning health systems framework used to analyse mortality data use in Mozambique’s health system (adapted from flagship report of the alliance for health policy and systems research, 2021)

The interview audio was transcribed into Portuguese by two experienced outsourced transcribers. The transcripts were coded in NVivo version 14 (QSR International, Inc. Australia). Immediately after the interviews, and throughout the transcription and data analysis, AM kept brief reflective memos to reveal prior assumptions, document decision points, and bracket emerging interpretations. Analysis was guided by the LHS framework (Fig. 2); an established approach to health systems that defines learning in three interconnected dimensions and allows the themes to be categorised according to:

- Learning levels (Individual: Health workers and managers; Organisational: Health facilities, MoH district/provincial; and Cross-organisational: Coordination across MoH, other sectors and NGOs);
- Loops of learning in the health system (Single-loop: improve practices without changing goals; Double-loop: Questioning and reviewing goals and policies; and Triple-loop: improve learning processes); and,
- Characterised by specific Means of learning (Information, Deliberation, and action).

First, the interview transcripts were coded deductively using a predetermined set of themes, followed by an inductive approach in which emerging themes from the data were incorporated into the initial framework [20]. Based on themes emerging throughout the coding process, the reading of interview transcripts, and an initial analysis, a second and final cycle of coding was applied (see Supplementary Material File S2: Annex II Codebook). The content of the transcripts, which had been coded in NVivo, was transferred to an Excel file designed through the lens of the LHS and pivot tables were used to create cross-tabulations and to summarise quote frequencies. This process leveraged the analysis process and the construction of narratives to answer the research questions. It supported the research by highlighting patterns and gaps, and by guiding the selection of illustrative quotes and contrasting cases.

## Results

### Respondents socio-demographic characteristics

We conducted twenty [20] key informant interviews with local key informants, of whom 16 were MoH officials (10 at the national level and 6 at the provincial level) and 4 local NGO representatives in Maputo and Zambézia Provinces. The interviewees’ socio-demographic characteristics are summarised in Table 1. The majority of respondents were staff with three or more years of experience in public health policy design and intervention.

**Table 1:** Characteristics of key informants interviewed about mortality data use in Mozambique’s health system, September–November 2024

Charateristic	Maputo	Manhiça	Quelimane	TOTAL
Site n (%)	10 (50%)	3 (15%)	7 (35%)	20 (100%)
Sex				
* Male*	6	2	4	**12 (60%)**
* Female*	4	1	3	**8 (40%)**
Education Level				
* Professional technician*	0	1	0	**1 (5%)**
* University/Higher*	10	2	7	**19 (95%)**
Participants category				
* Decision-maker*	6	1	3	**10 (50%)**
* Manager and health provider*	1	2	4	**7 (35%)**
* Researcher and Analyst*	3	0	0	**3 (15%)**
Years of Experience				
* 0–2*	4	0	2	**6 (30%)**
* 3–5*	4	2	3	**9 (45%)**
* 5 +*	2	1	2	**5 (25%)**
Institutional affiliation				
* Ministry of Health*	10	2	4	**16 (80%)**
* NGO*	0	1	3	**4 (20%)**
Administrative level				
* National*	8	0	0	**8 (40%)**
* Provincial*	2	0	5	**7 (35%)**
* District*	0	3	2	**5 (25%)**

At the MoH level, ten key informants in the position of National Directors (decision-makers) representing the Public Health sector were included, as well as seven managers and health providers representing the Provincial and District levels and NGOs. The remaining three key informants were analysts and research specialists at various levels.

### The overall relevance of mortality data in the health system

Table 2 summarises our findings through the LHS lens, highlighting the enablers and challenges to learning from mortality data across different levels of the health system. Participants stated that mortality data play a critical role in learning and decision-making processes within Mozambique’s health system. From the 20 key informant interviews, we coded 68 excerpts that span how mortality data are accessed, valued, used, and the factors that shape these processes (see Supplementary Material File S3: Annex III). Most of the quotes describe learning at the organisational level, underscoring the central role of the MoH as an institutional structure in interpreting and acting on mortality trends. Fewer narratives from individual and cross-organisational levels point to gaps in collaboration with partners and limited frontline empowerment to use mortality data.

**Table 2:** Enablers and challenges to learning from mortality data through a learning health system lens in Mozambique

Health System Level & Dominant Learning Loops and Means	Enablers	Challenges
◼ Individual level Single-loop learning via Information and Deliberation (case discussion and targeted changes).	Regular case reviews/discussions (maternal/perinatal death), generate action plans and immediate improvements of clinical practices.	—
◼ Organisational level (Subnational) Single-loop learning through Information. Deliberations are often not supported by concrete data, resulting in vague actions.	—	Untimely data & long production reduce relevance for current decision windows: they are perceived as “just another report”.
	Facility reports and meetings with mortality surveillance trigger rapid responses and actions.	Low data literacy among managers and decision-makers leads to poor data translation and prevents informed deliberation and strategic adjustment.
◼ Organisational level (National) Double-loop learning is dominant, although poor data translation prevails, hindering deliberation and strategic adjustment. Triple-loop learning was prevented.	Situational analyses: high-level planning forums (reprioritisation and resource reallocation); And leadership engagement to use DHIS2, and mortality surveillance data to monitor trends.	Fragmented systems; Multiple data sources with limited integration lead to gaps and competing truths. Limited data literacy among top-level decision-makers.
	—	Centralisation of decision-making processes narrows subnational decision space – limiting their autonomy and ability to act.
◼ Cross-organisational (MoH; NGOs; other sectors) Single/Double-loop learning to address changes. Superficial Deliberation.	Survey and Mortality surveillance data are integrated into planning meetings and cross-organisational discussions.	Fragmented and parallel data.

The results highlight a strong tendency toward double-loop learning, focusing on strategy re-prioritisation, resource reallocation and partner alignment. However, in several cases, the explicit use of mortality data was not evident. For example, participants reported high-level deliberation that aligns partners with national priorities to improve health outcomes, as one participant described: *“We contacted all the partner organisations that support the health sector and informed them of our priorities for 2025… We asked them to align their programmes with these plans*,* as this is the only way we will have the alignment necessary to achieve our objectives”* (Key informant 12, Decision-maker, Maputo). Single-loop learning is also evident, although less than double-loop, focusing on improving practices and actions, or small-scale activities within the health system without changing the assumptions, as a health provider mentioned the use of daily or weekly facility reports to trigger immediate responses to situations of maternal and neonatal death.

Meanwhile, cases of triple-loop learning are rarely reported by respondents, suggesting limited opportunities to challenge underlying assumptions to improve how the system learns, as one participant described: *“…the data is not timely. When the reports come out*,* they are just another report*,* because they are not aligned with decision-making needs*,* so we miss the opportunity to use them”* (Key Informant 07, Researcher and Analyst, Maputo). Information and deliberation were the most frequently reported means of learning when it comes to mortality data. Learning through information was the most cited pathway, reflecting narratives about the importance of accessing and analysing existing data to inform decisions at the MoH central level. Although less frequently mentioned, learning through action was visible during outbreaks and program adjustments at the subnational level. As a key informant noted, they focused on learning through practice, adapting protocols and reorienting activities in real time.

*“We had to make adjustments and redirect the program when we had the situation of increased cholera deaths in Nampula…”* (Key informant 13, Decision-maker, Maputo).

### Learning across levels

#### Individual level

At the individual level, some participants mentioned that healthcare providers use health facility mortality data to refine clinical practices and adapt patient-centred care, supporting continuous learning and improvement, which focuses on short-term adjustments to clinical services. In Manhiça, for example, providers reported regular maternal and neonatal death-review meetings that surface preventable factors and generate targeted action plans. These participants emphasised the multifaceted value of accessing facility mortality data, highlighting its influence on clinical practices.

*“There are monthly meetings to discuss maternal deaths and neonatal deaths… Then*,* depending on the conclusions we draw from the discussion*,* we have to take action and draw up an action plan to improve the service”* (Key informant 02, Health provider, Manhiça).

#### Organisational level

- Subnational and facility level

At the subnational and facility levels, participants mentioned that to understand the situation, they frequently have access to daily reports from health facilities, which they use to identify disease outbreaks and prioritise rapid responses. Here we see that deliberation occurs in ad-hoc huddles or district/facility meetings, and the action follows through with targeted responses and changes.

*“For example*,* the surveys don’t give us a cholera resistance situation. This is data that we need to have urgently to be able to act. Our monitoring tools*,* our record books from health facilities*,* give us this information*,* and we immediately rush to make decisions”* (Key informant 05, Manager, Quelimane).

Participants also emphasise that these rapid actions are possible thanks to administrative decentralisation within the health system.

*“…We now have administrative decentralisation*,* which helps us to look into the data and make decisions quickly at the local level”* (Key informant 02, Manager, Quelimane).

They also highlighted a trend towards data-sharing-based collaboration to intensify joint efforts at the subnational level. This facilitates discussions and enables interventions to be prioritised more effectively. This process reflects a stronger trend toward single-loop learning, but the emergence of double-loop learning through deliberation is also evident.

b) National level

At the national level, participants mentioned that they access all available data in the HIS to review health system performance, monitor trends and inform national planning. In some accounts, these processes went beyond routine monitoring and suggested elements of double-loop learning, particularly when mortality and other health indicators provided by the routine HIS prompted reconsideration of priorities, adjust strategic plans, or realign resource flows.

*“We’re looking at everything that DHS and SIS-MA [DHIS2] transmit in terms of the situation of the main indicators… We have learned what we must do to change the situation in the country”* (Key Informant 12, Decision-maker, Maputo).

Participants also emphasised that the use of routine HIS data is part of a dynamic, ongoing learning process at the central level of the MoH, providing a basis for strategic planning and resource allocation. However, most accounts described a sequence of case detection for action, with little detail on systematic monitoring that values the use of mortality data to measure the effect of actions and adjust the strategy.

*“…clearly yes*,* this has been going on continuously within the MoH*,* and we are making strong use of the data offered to us by SIS-MA [DHIS2] to adjust our actions”* (Key Informant 10, Decision-maker, Maputo).

Another participant pointed out that these data can also be used to strengthen national primary health care campaigns, including childhood immunisation.

*“…One area that we must focus on is strengthening primary health care*,* especially the issue of child vaccination… so*,* we use the data to orient these interventions”* (Key Informant 09, Decision-maker, Maputo).

Beyond the strategic reprioritisation and policy adjustments associated with double-loop learning, participants also described examples of transformative learning. In these cases, actors within the MoH sought to redesign decision-making processes and institutional learning structures, reflecting efforts to change not only what decisions are made, but also how the system learns and makes decisions. As one participant noted, *“Once we have understood the internal and external scenario*,* then we will say OK*,* these are the aspects we want to transform*,* and then we will initiate the theory of change”* (Key Informant 12, Decision-maker, Maputo).

#### Cross-organisational level

At the cross-organisational level, participants describe the extent to which learning collaboration between the central level of the MoH and the research institutions was critical to considering mortality data and its relevance in informing the development of the National Strategic Plan for the Health Sector.

*“The MoH is developing the next Strategic Plan for the next ten years. Mortality data has been brought to these forums because decision-makers recognise that it is important to have mortality information so that they can develop a plan focused on what really matters for public health… So*,* our mortality data is being used as one of the main tools for prioritising areas for the next Strategic Plan”* (Key Informant 04, Researcher and Analyst, Maputo).

Participants also highlighted that at the subnational level, periodic updates of mortality results in settings with active mortality surveillance have provided a concrete basis for discussions and deliberations among various stakeholders, including NGOs and other sectors.

*“We also participate in various forums where district and provincial mortality data are presented… this helps us to align our interventions”* (Key informant 08, NGO-Manager, Manhiça).

In this regard, a participant from the central level of the MoH added that when data is shared routinely and discussed in coordination forums, cross-organisational learning is stronger, partners align activities with MoH priorities and make coordinated strategic adjustments rather than isolated moves. As one participant noted, *“Above all*,* we have to ensure that the partners have access to all the data. They [NGOs] recognise our data and use it to design their intervention projects”* (Key informant 09, Manager, Maputo).

### Learning loops

#### Single-loop learning

Learning in its simplest single-loop form was most visible at the facilities and subnational levels, where discussions guide clinical practice performance. Learning was also evident in rapid responses, where teams used available data to make decisions about adapting and improving regular practices, and to address immediate gaps at the subnational level. In addition to case analyses that were directly translated into action plans to improve care, district managers described making rapid changes to specific practices or interventions, leading to short-term adjustments without altering the underlying goals.

*“Our monitoring tools and health facility records provide us with this information*,* and we immediately take action to resolve relatively straightforward day-to-day situations”* (Key Informant 13, Decision-maker, Maputo).

Participants’ reports also demonstrate the frequent use of available information for specific deliberations to achieve better health outcomes. They highlighted the emergency response to measles-related deaths in one province as an example of a single-loop learning dynamic focused on rapid action to contain the outbreak and meet the programme’s target.

#### Double-loop learning

Double-loop learning appears to be the most prevalent loop at the central level of the MoH. Within this learning loop, mortality data are used for deeper learning to inform priority and strategic decisions at the central level of the health system. Participants described ongoing efforts to consult available mortality data to understand the health situation, questioning whether current approaches are adequate to address contextual changes. At the national level, they also described how evidence and indicators provided by routine data are used to monitor trends, adjust strategies, and inform national planning. They also highlighted the partners’ efforts to align the process of reviewing health sector priorities as part of the joint learning loop. They emphasised the meaningful involvement of NGOs in government and health sector meetings. The resulting changes led to the creation of a joint platform where NGOs are asked to base their projects on the country’s health sector priorities.

*“…recently*,* senior management decided to hold a meeting with all organisations [NGOs]*,* and put the issue for discussion*,* with the same goal… What is the area of action within the platform? So*,* a lot of work has been done in this regard to try to redistribute everyone’s efforts according to priorities*,* so that we can achieve tangible results”* (Key Informant 10, Decision-maker, Maputo).

Participants from NGOs generally acknowledged that significant progress had been made in leveraging mortality data systems and in promoting collaboration with the MoH, thereby maximising access to and the potential of data for learning across different levels of the health system.

*“As partners NGO*,* we already have access to the systems*,* so we can log in and request data to inform our actions”* (Key informant 06, NGO-Manager, Quelimane).

Another participant from the MoH described actively accessing and using routine data to challenge primary health care priorities, moving beyond small adjustments to rethink what should be emphasised:

*“…One area that we must focus on is strengthening primary health care*,* especially the issue of child vaccination… so*,* we use the data to drive these interventions”* (Key Informant 12, Decision-maker, Maputo).

The above illustration shows that one of the most effective ways to leverage the potential of mortality data in the health system is through double-loop learning, highlighting its use to reexamine whether existing priorities were adequate for elevating primary health care and childhood immunisation as strategic objectives.

#### Triple-loop learning

Participants rarely described evidence of triple-loop learning. Instead, they highlighted several missed opportunities for the health system to learn and adopt new forms of learning. However, one participant at the highest level of the health system mentioned cases of transformative learning, in which MoH actors redesigned processes to enable changes in the learning context, thereby reshaping how the system makes decisions in various programmes. Additionally, participants described the persistence of a theory of continuous change involving extensive consultations as a potential step towards institutionalising learning within the health system.

*“We also consulted with civil society and the private sector because it’s in their interest too. And now it’s the provinces’ turn*,* and we are wrapping things up so that we can move on to the next steps*,* and develop the theory of change… Once we have understood the internal and external scenario*,* then we will say OK*,* these are the aspects we want to see*,* and we’ll design the theory of change”* (Key Informant 12, Decision-maker, Maputo).

At the same time, several narratives from participants highlighted the reasons why triple-loop learning remains largely invisible, with two of them standing out: externally preconceived projects that follow the agendas of international NGOs, and centralised authority that often prevents experimentation with new learning routines, limiting movement beyond underlying solutions and keeping triple-loop learning rare.

*“… sometimes when one program ends*,* another one starts with a different agenda*,* and sometimes we don’t even know what the ultimate goals are*,* what data they used to guide themselves. Sometimes they do their own research and use the data… then we miss opportunities to learn and improve some interventions in the field.”* (Key Informant 11, Manager, Maputo).

### Means of learning

#### Learning through information

Learning through information is highlighted by participants who describe accessing various sources of information, including facility records, mortality surveillance results, and survey results, to understand trends and identify priorities. Additionally, participants at the subnational level mentioned that, in places with active mortality surveillance systems, regular updates provided a concrete basis for joint interpretation at the district level.

*“We usually receive invitations to take part in meetings where this data on the mortality situation in our province is presented… we also discuss which areas we should prioritise according to the data”* (Key informant 05, NGO-Manager, Quelimane).

However, participants also highlighted limitations, namely timeliness and fragmentation, that weaken the informational foundation.

*“Unfortunately*,* in addition to the fragmentation of the system*,* some of this data reaches us a little late*,* which complicates our timely action”* (Key Informant 10, Decision-maker, Maputo).

#### Learning through deliberation

Deliberation was one of the dominant learning routes in health systems. It took place through dialogue, reflection, and review of clinical processes, district coordination meetings, integrated national planning sessions, and research dissemination events. According to participants, discussions in these forums enable them to learn from experience about the current situation, question current approaches, and sometimes support strategic changes within the health systems plan.

*“We’ve already finished the situational analysis*,* a look at the internal and external environment and we’ve planned a series of analyses of previous strategic documents*,* a series of interviews with different sectors to get a good understanding of the previous situation…”* (Key Informant 12, Decision-maker, Maputo).

Although participants considered that the use of mortality data in the health system is low, they emphasised that the use of routine HIS data is part of a dynamic, ongoing learning process at the organisational level, providing a basis for strategic planning and resource allocation. Another example of deliberative learning was the MoH Coordinating Council, which, according to one participant, brings together MoH actors at all levels, NGOs, and civil society to discuss the situation of maternal and child mortality in the country.

Research specialists and data analysts have pointed out that this type of deliberation was more prevalent when mortality data was shared at meetings with the MoH, reinforcing understanding and deep reflection on the evidence, influencing a change in approach that enables effective action.

*“…after the presentation of the results*,* there were many questions and discussions. Everyone was looking forward to a change of scenery regarding mortality. They showed that there is interest*,* and several suggestions were made to have tangible results about the mortality scenario in the country”* (Key Informant 04, Researcher and Analyst, Maputo).

#### Learning through action

Learning through action followed deliberations that led to routine and strategic learning, particularly at the subnational level. Participants reported that actions on the front lines included revised processes and the adoption of rapid local-level responses to outbreaks.

*“There were deaths in the community due to a measles outbreak*,* so we had to make decisions to prevent further deaths… we had to act and implement emergency intervention to solve the situation…”* (Key informant 09, Decision-maker, Maputo).

At the central level, actions included redefining priorities, reallocating resources, and redesigning programs, such as strengthening primary health care and child immunisation, which are then implemented at the subnational level. However, many participants’ reports described a sequence of detection and action with limited systematic follow-up. Due to their timely availability, mortality data were inconsistently used to monitor the effects of actions and recalibrate strategies, which weakened the learning cycle.

### Factors that shape the health system’s ability to learn from data

Participants described a set of systemic and interconnected factors that influence the ability of health systems at subnational and national levels to learn from mortality data. Four of these constraints were: untimely data; low data literacy among managers and decision-makers, fragmented information systems, and centralised decision-making that limits subnational autonomy and capacity to act.

At the national level, participants highlighted that delays in data production and dissemination render mortality data irrelevant for timely policy debates, learning, and decisions, as the data arrive after key policy and planning windows have closed. One participant noted that data produced by different mortality surveillance systems is often limited to satisfying academic research agendas rather than public health needs, leading to deliberation being supported by outdated data.

The lack of data literacy among both subnational managers and national decision-makers limits the ability to assess, interpret, and use evidence, hindering learning from mortality data and preventing effective decision-making. Consequently, mortality data are less likely to consistently support deliberation and strategic action, even though they are used in some instances for operational adjustments.

*“… The literacy of decision-makers regarding the understanding and use of data to inform concrete public health decisions is very low.”* (Key Informant 10, Decision-maker, Maputo).

The existence of fragmented and poorly integrated data sources creates competing truths, hindering the effective use of this data. According to participants, this leads to serious gaps in evidence-based planning and to duplication of efforts, undermining the information base and preventing deliberations from being supported by available data.

*“The fragmentation of information is what complicates things even more… we have several systems collecting mortality data*,* it’s not possible to consider all that data to inform decisions”* (Key Informant 12, Decision-maker, Maputo).

An additional factor affecting the effective use of data at the subnational level relates to the centralisation of decision-making processes. Participants noted the total dependence of the local level on the central directives, with local actors relying heavily on central guidance, which limits their autonomy and their ability to act, and prevents triple-loop experimentation involving new ways of working and learning.

*“… I would say that the local level is really inactive and dependent; it doesn’t make a move on its own initiative*,* missing out on many opportunities to learn for itself”* (Key Informant 07, Researcher and Analyst, Maputo).

## Discussion

In this case study, we investigated the extent to which mortality data are accessed and used to support learning and inform decision-making within the health system, using the LHS conceptual framework (reference). This framework provides a structured perspective on how knowledge is transformed into practice through three interconnected dimensions: learning levels, learning loops, and learning means. In terms of breadth across levels, the use of mortality data is concentrated at the central MoH and irregular at subnational/facility levels. In terms of depth across loops, those episodes of data use sometimes reach double-loop learning through information and deliberation, leading to re-prioritisation and strategy shifts within the health system. The double-loop learning not only involves accessing and using data to improve practices and system functions but also challenges underlying assumptions, strategies, and policies. However, we observe a clear gap in triple-loop learning, with the health system continuing to learn from the data provided by the information system, leaving little room for systemic change in the overall learning process. Looking at the means, information is produced but often untimely or fragmented, deliberation is common but not always evidence-grounded, and action is typically decoupled from systematic feedback. Through this LHS lens, our findings highlight four key insights: (1) overall utilisation of mortality data is low within the health system; (2) timely access and limited data literacy among managers and decision-makers constitutes the main barrier to effective use of mortality data; (3) mortality data are used differently to inform decisions across different levels of the health system; and (4) the use of mortality data is misaligned with the decentralisation of power and decision making in certain programmatic areas of thegovernment system in Mozambique.

Despite the recognised value of mortality data among managers and decision-makers, our findings demonstrate that access to and use of these data remain limited and uneven, especially at the subnational level, suggesting a weak culture of continuous learning using mortality data within the health system. An important exception to this is maternal and perinatal deaths, where global and national policies require routine reporting for auditing processes through the maternal and perinatal death surveillance and response (MPDSR) [21, 22]. When implemented as intended, MPDSR approaches a full learning cycle and can achieve double-loop learning by revisiting important changes in the health system [23]. However, consistent with our findings, the feedback component is uneven, with variable follow-up on action plans and limited dissemination and monitoring beyond facilities, which constrains broader system learning and the adoption of triple-loop learning, which involves improving the way the system learns by making deliberate changes to its learning structures and processes.

Beyond maternal and perinatal deaths, the routine use of mortality data remains far more limited, echoing the pattern observed in other LMICs, where improvements in data systems have not translated into widespread data use [6, 17, 24]. An exception is outbreak response. As our results revealed, during cholera surges, high mortality signals triggered rapid deliberation and immediate actions, a classic single-loop learning pattern. A second, emerging exception is under-five mortality beyond the neonatal period. With the Child Health and Mortality Prevention Surveillance (CHAMPS) program, producing cause-specific evidence, some provincial and national forums reported new deliberations on referral quality, community case management, and vaccine or campaign targeting [15, 25], hinting at incipient double-loop use at the centre.

Meanwhile, global health initiatives have prioritised strengthening mortality surveillance and CRVS systems to produce more comprehensive mortality information [26, 27]. As a result, many countries with historically poor-quality mortality data and low completeness of mortality registration have invested heavily in advanced mortality surveillance tools and data collection systems [24, 28, 29]. Despite significant efforts to gather mortality data, there have been no corresponding efforts to promote the systematic integration of these data into decision-making processes. This gap is evident in our findings, which confirm that low overall use of mortality data reflects a broader pattern in which routine data, including DHIS2, facility reports and surveys, are consulted episodically, while capacity, incentives and accountability for sustained use remain weak [30, 31]. Therefore, strengthening utilisation requires coupling ‘data-supply’ upgrades with demand and use-side reforms: analytic capacity building, clear decision rights and timelines, feedback loops, and partner alignment.

With regards to timely access to mortality data, our findings suggest that untimely data, low data literacy among decision-makers, fragmented information systems, and weak alignment with partners are the main obstacles to its effective use, despite ongoing technical improvements in the health information infrastructure [6, 18]. Consistent with reports from other LMIC contexts [32], these challenges highlight a persistent gap between data availability and data usability. Mortality data are increasingly produced through routine health information systems, surveillance platforms, and surveys, yet availability alone does not ensure use. For data to inform decisions, they must reach the right actors at the right time, in a format that matches the decision cycle in question, whether annual planning, budgeting, routine performance review, or outbreak response. Our findings suggest that mortality data often fail to meet this standard. Although platforms like DHIS2 have enhanced data quality and availability, our analysis shows that decision-makers still require capacity to interpret basic statistics and trends, understand uncertainty and data limitations, compare indicators across places and time, and translate mortality patterns into operational priorities. Strengthening data literacy, therefore, requires more than one-off training; it calls for routine, hands-on support embedded in planning, review, budgeting, and outbreak-response forums where data are actually used [7]. Without these capacities and decision routines, the return on investment in data production is diluted, and datasets are often misinterpreted, rendering them unable to influence decisions. This raises an important value-for-money concern, as investments in mortality data generation have limited impact if they are not matched by mechanisms that support interpretation, deliberation, and action.

This data-usability gap is also important in light of the rapidly changing landscape of digital health, analytics, machine learning (ML), and large language model (LLM)-based decision support for public health decision-making [33, 34]. These tools may change how health systems learn from mortality data by making information more timely, accessible, and understandable. For example, ML-based tools, including digital and automated contact-tracing models, may help detect signals earlier, classify risk, and guide targeted outbreak responses [33, 35]. Applied to mortality surveillance, similar approaches could support earlier detection of mortality anomalies, outbreak intelligence, and decision-support products such as dashboards, alerts, or short decision briefs. LLM-based approaches may also help synthesise complex evidence, retrieve relevant guidance, and translate technical outputs into more accessible summaries for decision-makers [34]. However, our findings show that technical tools alone are insufficient beyond the available platforms and data. If decision-makers lack the capacity to understand and interpret outputs, if information systems remain fragmented, or if local actors lack authority and resources to respond, AI-generated insights may become another underused data product. Therefore, digital and AI-supported decision tools should be embedded within governance mechanisms that strengthen data literacy, interoperability, deliberation, accountability, and the decision-making process.

In Mozambique, initiatives such as the National Evaluation Platform (NEP) were introduced to strengthen institutional capacity to appraise and use evidence in strategic decisions. However, their integration into the routine functioning of the health system remains limited [3, 6]. This reinforces our broader argument that data-use reforms must address both technical and institutional conditions and must be communicated in formats that are usable for non-technical decision-makers. Practical approaches include simple dashboards, short policy or decision briefs, visual trend summaries, clear explanations of uncertainty and data limitations, and routine interpretation meetings where analysts and non-technical decision-makers jointly discuss implications for planning. As digital tools expand, ML and LLM-based approaches may improve the timeliness, processing, and visibility of mortality data, as well as support robust data interpretation. However, LLM-based tools also introduce risks, including hallucinated or poorly grounded evidence. Retrieval-augmented generation and GraphRAG approaches may help reduce these risks by grounding outputs in trusted documents, knowledge bases, and verified data sources. Even so, these tools are likely to have limited impact unless embedded within institutional mechanisms that connect information to deliberation and action. Otherwise, the LHS cycle stalls at the handover from information to deliberation, resulting in sporadic learning and limited system-wide improvement.

Interestingly, some common barriers reported in other similar contexts, such as poor data quality [36], were not as evident in our study, likely due to recent investments in Mozambique’s health information infrastructure and the implementation of the Sample Registration System (SRS), which has enhanced the consistency, quality and availability of mortality data. At the same time, beyond technical factors, the political economy of data can still restrict its use [37]. Population datasets (e.g., DHS and censuses) may be under-shared or selectively cited when trends are unfavourable, as actors fear reputational costs or funding repercussions [38]. This politicisation discourages transparent disclosure and open debate, mitigating reflection and any movement toward triple-loop change [39]. Although not a dominant theme in our study, this dynamic is consistent with broader evidence and deserves explicit attention in efforts to strengthen learning from mortality data.

On the other hand, fragmented data systems were reported as an additional barrier in both our findings and in previous studies [19, 40]. In Mozambique, fragmentation appears to stem not only from the number of systems, but also from weak alignment between MoH platforms, partner-supported tools, and donor reporting requirements [41]. In addition, these parallel channels can create uneven access to mortality evidence, duplicate reporting, and missed opportunities for district-level interpretation and action. This creates a tension where donors and NGOs provide essential technical, financial, and analytic support, but externally funded systems may also shape data priorities around project-specific indicators rather than national HIS priorities or district learning needs [42, 43]. Preserving partner support therefore requires stronger MoH-led governance, not the withdrawal of partner investment. A national data-governance compact should require that partner-funded data activities are co-designed with MoH, use SIS-MA/DHIS2 as the national backbone where feasible, shared data standards, clear rules on data access and ownership, and routine feedback and joint review meetings with provincial and district teams. Without these mechanisms, partner support may continue to generate useful data but fail to strengthen the LHS cycle, so the information will remain fragmented, deliberation superficial, action delayed, and feedback loops broken.

Looking across health-system levels, this study reveals marked differences in how mortality data are used. At the subnational level, mortality signals primarily drive operational, short-cycle decisions; a single-loop pattern in which routine reports and outbreak tallies trigger immediate actions such as targeted vaccination, water chlorination, and case management during outbreaks [27, 44].

In contrast, at the MoH level, mortality data are used to support medium-term planning and strategic adjustments, including alignment with national health priorities and resource allocation. In some cases, these processes suggest emerging double-loop learning, particularly where data were used to reconsider priorities, question whether existing approaches were adequate, or redirect programme strategies. However, the evidence did not always allow us to determine whether these central-level adjustments challenged underlying assumptions, goals, or policy logics, or whether they reflected high-level implementation adjustments within existing priorities. We therefore interpret national-level data use as a mix of strategic adjustment and emerging double-loop learning, rather than as uniformly double-loop. As noted in the adaptive management literature, in this situation, double-loop learning involves re-prioritisation and resource shifts as underlying assumptions are revisited [45]. Although stakeholders participate in reflective learning to develop a theory of change, these practices remain centralised and have not yet evolved into organisation-wide learning routines, underscoring the need for broader participation involving provincial services and NGO partners. This limits local action and prevents triple-loop learning experiences involving new ways of working, which would require a fundamental reshaping of how the health system learns, operates and adapts.

The prominence of double-loop learning at the organisational level highlights a reflective culture, but one that remains confined to central MoH actors. This observation aligns with previous research in sub-Saharan African countries, which describes a predominantly vertical decision-making structure, in which central-level decisions are implemented passively at subnational and local levels [24, 46]. In Mozambique, this reflects the persistence of a top-down governance model [47], which constrains the capacity of actors at the subnational level to engage meaningfully with mortality data and tailor their responses to local realities [40], despite the recent reforms that project decentralisation of power to the subnational level [48]. As a result, mortality data remain underused, even as overall data quality and availability improve, mainly because mortality surveillance systems collect and report local-level mortality trends. This suggests that decentralisation, on its own, is insufficient. Formal decentralisation must be matched by functional decentralisation, including discretionary budgets, technical capacity, and accountability mechanisms that enable local actors to act. Without these conditions, decentralisation risks reinforcing a technocratic model in which subnational actors comply with central reporting requirements rather than using data for local learning, adaptation, and performance improvement [49].

The continued concentration of data use at the central level, despite the presence of mortality surveillance systems in some districts, suggests that the main constraint is not just data availability alone. Rather, subnational use of data is limited by a combination of technical capacity, institutional culture, budget authority and political accountability [6, 47, 49, 50]. Although some decisions can be taken at the subnational level, most resources remain under national control, limiting the ability to translate local mortality data into timely action [47, 49]. This reinforces a pattern in which local-level data feed more into upward reporting and central planning than into learning and adaptation at the subnational level. Furthermore, some subnational mortality surveillance systems in Mozambique are designed primarily around research objectives and timelines. While these systems generate high-quality evidence, their outputs may not always be produced in a form or timeframe that supports routine public health decision-making. Subnational-level managers require timely, simplified and actionable information that aligns with planning, budgeting, performance review, and outbreak-response cycles. When local mortality data are collected primarily for academic purposes, compiled for dissemination in research forums, and reported to higher authorities, they are less likely to support routine learning and adaptation at the subnational level. To leverage the value of mortality data more effectively, reforms should therefore combine capacity building with clearer local decision-making authority, regular feedback loops, timely decision briefs, and incentives for subnational teams to use mortality evidence in planning, budgeting, and resource allocation. If systematically addressed, these shifts could reposition mortality data not only as a tool for monitoring outcomes but also as a foundation for a more adaptive, responsive, and learning-oriented health system.

This qualitative study has notable strengths and some important limitations that warrant consideration. By applying the LHS framework to explore how mortality data are used across different levels of Mozambique’s health system, the study offers relevant insights into how learning processes can be strengthened to drive meaningful improvements in health service delivery and outcomes. Qualitative design enabled a nuanced understanding of the contextual and institutional dynamics influencing learning, while the inclusion of perspectives from both national and subnational actors provided a more comprehensive view of data use practices across the health system. This multi-level representation adds depth to the analysis of how different learning loops function in practice. However, some limitations must be acknowledged. The study is based on self-reported data, which may be subject to social desirability bias and skewed by the experiences of participants who, despite occupying relevant positions in the health sector and other organisations, may not fully reflect different angles of reality. In addition, site selection may bias the findings, as Manhiça and Quelimane have long-standing mortality surveillance activities and relationships with Manhiça Health Research Center (CISM) and a stronger research culture than other regions, which likely increases awareness of, access to, and norms around data use. Therefore, our findings should be interpreted with attention to transferability. If barriers to mortality data use remain evident in settings with relatively abundant data and external support, then these barriers are likely equal to or more severe in districts without established mortality surveillance systems. Yet the study revealed important gaps and missed opportunities despite the privileges enjoyed by the two sites. It is also important to note that the absence of direct observation limits our ability to assess how mortality data are used in real-time decision-making. Lastly, while the LHS framework provided a valuable analytical lens, its potential application for a general understanding of learning from mortality data in the context of LMICs still requires adaptation to reflect local realities and the constraints on action and decision-making imposed by the system.

## Conclusion

This study describes how mortality data are accessed, interpreted, and used for learning and decision-making across different levels of the Mozambican health system. By identifying key learning patterns, including the dominance of double-loop learning through information, the study shows that although the overall use of mortality data remains relatively low, its application helps improve certain clinical practices at the individual level, strengthen health system functions at the organisational and cross-organisational levels, and challenge underlying assumptions, strategies, and policies. However, the absence of triple-loop learning and ongoing barriers to the use of mortality data highlight critical challenges to building a robust and inclusive learning culture, particularly at the subnational level. While progress has been made in recognising the value of mortality data, a more transformative shift is needed in how the health system learns, coupling “data-supply” upgrades with demand- and use-side reforms. This requires improving data accessibility, building analytical capacity, establishing structures that accommodate decentralised decision-making, aligning learning processes, and promoting the effective use of mortality data across all levels in the health system.

Furthermore, Mozambique can move from episodic analysis to routine learning by embedding time-bound mortality-driven feedback loops, aligning partner systems to the national HIS, and pairing decentralised authority with accountability.

## Supplementary Information

Supplementary Material 1.

Supplementary Material 2.

Supplementary Material 3.

## Data Availability

The datasets used and/or analysed during the current study are available from the corresponding author upon reasonable request.

## Abbreviations

CRVS: Civil registration and vital statistics system
CHAMPS: Child Health and Mortality Prevention Surveillance
HIS: Health information systems
LHS: The Learning Health System
LMIC: Low- and middle-income countries
MoH: Ministry of Health
MSS: Mortality surveillance systems
NGO: Non-governmental organisation
NEP: National Evaluation Platform
SSI: Semi-structured interviews
