# Supplementary material for: The value of mortality data to advance learning and decision-making dynamics in Mozambique’s health system
Source: Arch Public Health. 2026 Aug 14;84:243. doi: 10.1186/s13690-026-02044-5 (PMC13753472; doi:10.1186/s13690-026-02044-5)
Supplement: Supplementary file 1 [file 13690_2026_2044_MOESM2_ESM.pdf]

MANHIÇA HEALTH RESEARCH CENTRE (CISM)
The contribution and use of mortality data to inform health interventions in health facilities and communities in Mozambique

IN-DEPTH INTERVIEW GUIDE
Key Informants

Instructions

1. Explain to the participant that:

   The purpose of this interview is to explore how data on under-five mortality generated by different projects contribute to informing resource allocation, health planning and the implementation of strategies to reduce child mortality in Mozambique, particularly in the districts of Manhiça and Quelimane.

- You have been invited to this conversation or interview because we believe you have experience in this area and hold important information, so we think your contribution is essential to our understanding of the topics described above.

- Let's start by talking about your understanding of how mortality data is organised in Mozambique and how this data is used to specifically define public health interventions and policies for the context of your province/district. Then we will talk about your opinion on how this data should be explored and used to reduce child mortality in your province/district. This conversation is expected to last between 30 and 45 minutes, and no more than 1 hour. However, the time will depend on how the conversation progresses.

2. Say that, if they agree, the interview will be recorded, just so that we don't run the risk of losing any important information that comes up during the conversation. All recorded information will be confidential and you will not be identified by name. Also say that he may refuse to allow the conversation to be recorded. And that in all cases (whether he agrees to be recorded or not) you (the interviewer) will take notes throughout the conversation. Also say that we record the conversation and take notes throughout to ensure that we capture all the information he provides.

DEMOGRAPHIC INFORMATION

**Ref. Audio File / Study Number**	IDI_|__|__|-|__|__|__|__| - |__|__|__|__|__|__|__| (Interview ID) (File ID) (Participant ID - No. and 3 letters)	
**Participant data**	Gender |__ | Age |__|__ | Place of birth (province and town): ____________________<br>____________________<br>Residence (Administrative district and neighbourhood): ____________________<br>Level of education (last level completed): ____________________Marital status: ____________________<br>Institution: ____________________<br>Profession/Occupation: ____________________Length of time working in this role (years/months)__________<br>Religion and church: ____________________	
**Date and place of interview**	|__|__|/|__|__|/|__|__|__|__| ____________________	
**Languages spoken**	____________________	
**Interview result**	Recorded☐ Not recorded ☐ Reason:____________________<br>____________________<br>Complete ☐ Interrupted ☐ Reason: ____________________<br>____________________	Impossible to complete ☐<br>To be completed by (date):<br>____________________
**Interviewer:** |__|____|__|		

1. PERCEPTIONS AND PERSPECTIVES

	SUMMARY
1. Can you describe your main tasks and responsibilities since you took on this role? (Ask about length of service in the area)<br><br>2. Have you had opportunities to contribute to the development of public health policies or programmes/interventions that have been implemented in our country/province/district?<br><br>**Probe:**<br>➔ Can you describe that experience?<br>➔ What was your role and level of involvement?<br>➔ What programmes/interventions did you contribute to?<br><br>3. What is your opinion on the current state of children's health in Mozambique or in your province/district?<br><br>4. Do you think there have been changes in the health status of children over the last 10 years?<br><br>**Probe:**<br>➔ If so, what kind of changes have you observed? Why do you think these changes have occurred?<br>➔ If not, why do you think there have been no changes?<br>➔ What challenges and/or barriers have prevented change?	

5. What do you consider to be the priority issues in addressing children's health problems in Mozambique?<br><br>6. What is your opinion on the use of mortality data to inform health policies?<br><br>7. What is your perception of the reliability of existing mortality data in Mozambique?<br><br>**Probe:**<br>➔ Do you think this data is used to inform health policies?<br>➔ In what way?<br><br>8. What sources of data on child mortality do you regularly use or rely on?<br><br>**Probe:**<br>➔ Data from the health system/demographic health surveys/census/NGOs/health and mortality surveillance?<br><br>9. How does this data reach you?<br><br>**Probe:**<br>➔ Who requests the data?<br>➔ Who analyses the data for you?	

➔ How is the data analysed?	

DECISION-MAKING PROCESS

1. How do you use mortality data?<br><br>2. What specific aspects of children's health planning are influenced by mortality data?<br><br>3. Can you explain how the data is integrated into the planning process?<br><br>4. How is child mortality data translated into actionable strategies?<br><br>5. What challenges, opportunities, and successes are experienced during the implementation of strategies based on mortality data?<br><br>6. Are there factors that prevent the effective use of mortality data in resource allocation or planning?	**SUMMARY**

**Probe:**<br>➔ What are these factors?<br>➔ What causes this impediment?<br>➔ What should be done to improve or overcome the obstacles?<br><br>7. What are the other current or past obstacles that prevented the development and implementation of specific strategies/programmes and/or policies for children's health?<br><br>**Recommendations for improvement:**<br><br>8. What recommendations do you have for optimising the contribution of data to health decision-making?<br><br>9. Is there anything we haven't discussed today that you would like to share on this topic?	

COMMENTS:
