# Supplementary material for: The value of mortality data to advance learning and decision-making dynamics in Mozambique’s health system
Source: Arch Public Health. 2026 Aug 14;84:243. doi: 10.1186/s13690-026-02044-5 (PMC13753472; doi:10.1186/s13690-026-02044-5)
Supplement: Supplementary file 2 [file 13690_2026_2044_MOESM1_ESM.docx]

**Supplement material III_ S3:**

**Annex III:** Overview of results across levels, loops, and means of learning within the health system

| **Learning Loops and Levels in the Health System** | **MEANS OF LEARNING** | | | |
| --- | --- | --- | --- | --- |
|  | **Information** | **Deliberation** | **Action** | **Grand Total** |
| **Access of mortality data in the Health System** | **10** | **2** | **-** | **12** |
| **I. Single** | **3** | **1** | **-** | **4** |
| Organizational | 3 | 1 | - | 4 |
| **II. Double** | **7** | **1** | **-** | **8** |
| Organizational | 6 | 1 | - | 7 |
| Cross organizational | 1 | - | - | 1 |
| **Value of mortality data in the Health System** | **14** | **4** | **4** | **22** |
| **I. Single** | **6** | **1** | **1** | **8** |
| Individual | 1 | 1 | - | 2 |
| Organizational | 4 | - | 1 | 5 |
| Cross organizational | 1 | - | - | 1 |
| **II. Double** | **8** | **3** | **3** | **14** |
| Organizational | 8 | 2 | 3 | 13 |
| Cross organizational | - | 1 | - | 1 |
| **Use of data for Decision-Making** | **9** | **7** | **6** | **22** |
| **I. Single** | **-** | **2** | **3** | **5** |
| Individual | - | 1 | - | 1 |
| Organizational | - | 1 | 3 | 4 |
| **II. Double** | **9** | **4** | **3** | **16** |
| Organizational | 9 | 3 | 2 | 14 |
| Cross organizational | - | 1 | 1 | 2 |
| **III. Tripple** | **-** | **1** | **-** | **1** |
| Organizational | - | 1 | - | 1 |
| **Factors that shape the health system's ability to learn from data** | **9** | **3** | **-** | **12** |
| **I. Single** | **4** | **2** | **-** | **6** |
| Organizational | 2 | - | - | 2 |
| Cross organizational | 2 | 2 | - | 4 |
| **II. Double** | **3** | **1** | **-** | **4** |
| Individual | 1 | - | - | 1 |
| Organizational | 2 | 1 | - | 3 |
| **III. Tripple** | **2** | **-** | - | **2** |
| Organizational | 2 | - | - | 2 |
| **Grand Total** | **42** | **16** | **10** | **68** |
