# Supplementary material for: The value of mortality data to advance learning and decision-making dynamics in Mozambique’s health system
Source: Arch Public Health. 2026 Aug 14;84:243. doi: 10.1186/s13690-026-02044-5 (PMC13753472; doi:10.1186/s13690-026-02044-5)
Supplement: Supplementary file 3 [file 13690_2026_2044_MOESM3_ESM.docx]

**Project 1_ Obj4_ Contribution of mortality data for learning and decision making in the health system**

Nodes

| **Name** | **Description** | **Files** | **References** |
| --- | --- | --- | --- |
| (Inf. Category 1) Value and utility of Mortality Data | All information related to access and use of mortality data to inform health actions and interventions. | 1 | 1 |
| Challenges | Challenges in access and use of data by decision-makers | 10 | 23 |
| Accessing data |  | 3 | 7 |
| Using data |  | 4 | 6 |
| I. Double Loop |  | 5 | 8 |
| I. Single Loop |  | 3 | 4 |
| I. Tripple Loop |  | 0 | 0 |
| Interventions informed by mortality data | Interventions informed by mortality data | 1 | 2 |
| Relevance of mortality data | Participants' opinions on the importance/relevance of mortality data in informing public health actions | 8 | 13 |
| (Inf. Category 2) Resource Allocation | All information related to the processes that influence the allocation of resources for health/public health interventions | 0 | 0 |
| Challenges for effective resource allocation |  | 5 | 7 |
| Coordination among MoH and Stakeholders | Coordination between MISAU and stakeholders in resource allocation. | 7 | 18 |
| II. Double Loop |  | 3 | 4 |
| II. Single Loop |  | 4 | 5 |
| II. Tripple Loop |  | 1 | 1 |
| Processes that influence resource allocation | What processes influence the allocation of resources (financial, HR, infrastructure) in the health sector? | 2 | 3 |
| Resource Allocation |  | 0 | 0 |
| (Inf. Category 3) Integration of data into Strategic planning in the health sector | All information that influences procedures and strategic planning in the health sector | 0 | 0 |
| Decentralisation and local strategies | All information related to decentralisation and the implementation of strategies at local level. | 7 | 14 |
| Strategies and contextualised interventions |  | 3 | 8 |
| III. Double Loop |  | 3 | 7 |
| III. Single Loop |  | 0 | 0 |
| III. Tripple Loop |  | 0 | 0 |
| Priorities to the Strategic Plan | Information related to priorities in the design of strategic plans for the health sector | 2 | 6 |
| Type of Data used to inform Strategic planning | What data is used to inform strategic plans for the health sector | 3 | 5 |
| The role or value of mortality data | The role of mortality data in strategic planning | 4 | 5 |
| (Inf. Category 4) Use Data for Planning and Decision-Making Processes | All information related to the decision-making process in the health sector | 0 | 0 |
| IV. Double Loop |  | 5 | 6 |
| IV. Single Loop |  | 5 | 6 |
| IV. Tripple Loop |  | 1 | 1 |
| Translating data to policy information | All information related to participants' perceptions of the translation of mortality data into policy information. | 0 | 0 |
| Challenges for contextualized interventions (Local level) |  | 4 | 5 |
| Data for Decision-making at Local level (Decentralization) | Decentralisation and use of data for decision-making at the local level (provincial/district). | 8 | 13 |
| Data for Decision-making at National level |  | 1 | 1 |
| 5. Recommendations_ Suggestions for improving the use of mortality data | All suggestions for improving the way mortality data is accessed and used by public health decision-makers | 8 | 14 |
